# COVID-19 vaccination remains essential for healthcare professionals: clinical evidence from a Polish cross-sectional study

**DOI:** 10.3389/fpubh.2026.1750819

**Published:** 2026-04-16

**Authors:** Karolina Hoffmann, Michał Michalak, Marzena Jarząb, Manfredi Rizzo, Sorina Ispas, Ioannis Ilias, Viviana Maggio, Mohammed El-Tanani, Syed Arman Rabbani, Sirajunisa Talath, Imran Rashid Rangraze, Walaa Ibraheem, Shakta Mani Satyam, Adil Farooq Wali, Bhoomendra Bhongade, Ashot Avagimyan, Anna Paczkowska

**Affiliations:** 1 Department and Clinic of Internal Diseases and Metabolic Disorders, Poznan University of Medical Sciences, Poznań, Poland; 2 Department of Computer Science and Statistics, Poznan University of Medical Sciences, Poznań, Poland; 3 Department of Pharmacoeconomics and Social Pharmacy, Poznan University of Medical Sciences, Poznań, Poland; 4 Promise Department, School of Medicine, University of Palermo, Palermo, Italy; 5 Ras Al Khaimah Medical and Health Sciences University, Ras Al Khaimah, United Arab Emirates; 6 Department of Anatomy, Faculty of Medicine, Ovidius University, Constanta, Romania; 7 Department of Endocrinology, Hippokration Hospital, Athens, Greece; 8 Department of Pharmaceutical Chemistry, Ras Al Khaimah Medical & Health Sciences University, Ras Al Khaimah, United Arab Emirates; 9 Department of Clinical Pharmacy and Pharmacology, RAK College of Pharmacy, RAK Medical and Health Sciences University, Ras Al Khaimah, United Arab Emirates; 10 Department of Pharmacology, RAK College of Medical Sciences, RAK Medical and Health Sciences University, Ras Al Khaimah, United Arab Emirates; 11 Department of Internal Medicine, RAK College of Medicine, RAK Medical and Health Sciences University, Ras Al Khaimah, United Arab Emirates; 12 RAK College of Pharmacy, RAK Medical & Health Sciences University, Ras Al Khaimah, United Arab Emirates; 13 Department of Internal Diseases Propedeutics, Yerevan State Medical University After M. Heratsi, Yerevan, Armenia

**Keywords:** COVID-19 health consequences, COVID-19, healthcare professionals, SARS-CoV-2, vaccination

## Abstract

**Introduction:**

Six years after the emergence of SARS-CoV-2, vaccination remains still central to preventing severe COVID-19. The aim was to determine the impact of the vaccine type and number of doses on the course of COVID-19 infection among Polish healthcare professionals.

**Methods:**

From November 2021 to February 2022, 813 people took part in a survey, including 625 women (76.9%) and 188 men (23.1%).

**Results:**

743 respondents (91.39%) had no confirmed COVID-19 infection after taking one of the available vaccines. However, there was no significant relationship between the number of doses of the SARS-CoV-2 vaccine and the occurrence of coronavirus infections after their administration (*p* = 0.75). Among people vaccinated with three doses, COVID-19 was mild (75.92%). Only 2.86% of the vaccinated subjects required hospitalization due to COVID-19 vs. 30.76% among unvaccinated subjects (*p* < 0.0001).

**Conclusion:**

The type of preparation taken did not affect the re-infection with the coronavirus, its course and the risk of complications. Vaccinated people were significantly less likely to be hospitalized due to COVID-19. There was no significant relationship between the number of doses of the SARS-CoV-2 vaccine and the occurrence of COVID-19 after their administration.

## 1 Introduction

It has now been 6 years since the first cases of COVID-19 were detected. On December 31, 2019, for the first time, WHO officially confirmed a case of infection with a disease caused by SARS-CoV-2. On January 13, 2020, a case of a person with COVID-19 was confirmed in Thailand, and on January 29, 2020, sick persons were diagnosed in all provinces of China. On January 30, 2020, the WHO Director-General assessed the situation related to the SARS-CoV-2 virus as PHEIC (Public Health Emergency of International Concern), i.e., a public health threat of international concern. On March 11, 2020, COVID-19 was officially defined as a pandemic (1–4).

In Poland, the first case of infection with the SARS-CoV-2 virus was diagnosed on March 4, 2020. In accordance with the regulation of the Minister of Health, the state of epidemic was introduced that enabled anti-epidemic and preventive measures aimed at minimizing the effects of the epidemic, e.g., various restrictions, the obligation to quarantine and isolate in case of COVID-19 (5, 6).

The available pharmacotherapy was not able to stop the pandemic. The only effective option in the fight against COVID-19 were vaccines. Nowadays, it is estimated that over 13.3 billion vaccine doses have been used in the global population (7). The struggle with SARS-CoV-2 became successful and on May, 5, 2023 the head of the UN WHO ‘has declared “with great hope” an end to COVID-19 as a public health emergency’ (7).

The presented study was conducted between November 2021 and February 2022, after 10 months of The Polish National Vaccination Program (8). The project was initiated in order to evaluate the effectiveness of COVID-19 vaccines in the population of Polish healthcare professionals and medical students.

The vaccination of Polish medical staff (the so called ‘zero’ group) had started on December 27, 2020. Precisely, the so called ‘zero phase of vaccination’ was primarily healthcare workers who were most at risk of contracting the coronavirus. The zero stage of vaccination involved not only doctors, but also diagnosticians, pharmacists, psychologists, support, technical and administrative staff in hospitals and medical facilities (including employees of private healthcare providers). At this stage, the vaccine was also administered to academic teachers and students of medical faculties (8).

Poland, as a member of the European Union (EU), has adopted a strategy of joint purchasing and distribution of vaccines, which is based on ensuring supplies to the Member States due to contracts concluded in advance with producers (9). Between November 2021 and February 2022, there were five vaccines approved for marketing in Poland (10):

(1) Comirnaty mRNA vaccine (BNTI162b2) from Pfizer/BioNTech;
(2) Spikevax mRNA vaccine (mRNA 1,273) from Moderna;
(3) Vaxzevria vector vaccine (ChAdOx1 nCoV-19) from Oxford/AstraZeneca;
(4) ‘COVID-19 Vaccine Janssen’ vector vaccine (Ad26. COV.2-S), later name: Janssen Jcovden (Johnson & Johnson);
(5) Nuvaxovid protein vaccine (NVX-CoV2373) Novavax.

Two vaccines (Comirnaty and Spikevax) are mRNA vaccines in the form of lipid nanoparticles in which a template ribonucleic acid modified with nucleosides is enclosed (11–15). Alternatives are vector-based vaccines: Vaxzevria vaccine and Vaccine Janssen COVID-19 vaccine. They contain recombinant adenoviral vectors that encode the characteristic spike glycoprotein (Spike protein) of the SARS-CoV-2 virus (16). On January 13, 2022, the European Commission approved the Nuvaxovid protein vaccine by NOVAVAX. It consists of purified full-length recombinant SARS-CoV-2 virus S proteins, stabilized in the pre-fusion conformation (17, 18).

The introduction of a vaccination program against COVID-19 was an indispensable element of effective prevention of COVID-19. Vaccines prevent severe illness, hospitalization and death. According to clinical trials conducted by Pfizer, the Comirnaty mRNA vaccine showed the effectiveness of preventing SARS-CoV-2 infection at the level of 91.3% of subjects, but after 4 months it decreased to 77% (16, 19).

Moderna’s mRNA vaccine, at the time of phase 3 clinical trials being the basis for conditional marketing authorization, showed 94.1% effectiveness in preventing infection in the study population (11). According to the Institute for Health Metrics and Evaluation (IHME), the Spikevax vaccine prevents Delta mutation infection by 91% and severe symptoms by 97%. It protects against infection with the SARS-CoV-2 virus with the Omicron variant in 48%, and in 73% it prevents severe course (20). The Vaxzevria vaccine was also successful in preventing COVID-19 at 76% after the first dose, and after taking the second dose at the recommended interval of at least 12 weeks, the effectiveness increased to 82% (21). COVID-19 Vaccine Janssen was conditionally approved for marketing, showing an effectiveness in protection against COVID-19 symptoms at the level of 67 and 77% against severe course of the disease (22, 23).

It should be emphasized that the period of clinical trials was insufficient, phase 2 and 3 trials were conducted simultaneously, that EMA allowed these vaccines to be marketed conditionally, which means that clinical trials are still ongoing, once a year, vaccine manufacturers must provide EMA with new scientific evidence on the basis of which the summary of product characteristics (SmPC) is updated (23). For this reason, the effectiveness of these vaccines needed to be evaluated in clinical practice, and the authors of presented study made an attempt to do it.

Current Polish recommendation states that healthcare workers in direct contact with patients or long-term care residents are among those for whom vaccination is especially indicated (24). Despite the growing body of literature on COVID-19 vaccine effectiveness, several important gaps remain. First, there is limited real-world evidence focusing specifically on healthcare professionals and medical students in Central and Eastern Europe, particularly in Poland. Second, most available studies assess selected vaccines or outcomes separately, whereas our study provides a comprehensive evaluation of multiple vaccine types administered within the same population. Third, our analysis simultaneously considers infection rates, disease severity, complications, and hospitalization risk, offering a broader clinical perspective. Finally, the study reflects a specific epidemiological period dominated by the Delta variant, which allows for context-specific interpretation of vaccine effectiveness. Therefore, our findings contribute novel and region-specific evidence to the existing literature.

Taking into consideration the above-mentioned facts, this study aimed to evaluate the effectiveness of COVID-19 vaccines among Polish healthcare professionals and medical students, with particular emphasis on the impact of vaccine type and number of doses on the course of COVID-19 infection.

## 2 Materials and methods

### 2.1 Sample composition

This post-marketing trial was designed as a cross-sectional survey-based study. Participants were recruited nationwide from among healthcare professionals (doctors, pharmacists, and nurses) as well as medical students in Poland. Five thousand e-mails were sent to the above healthcare workers and medical students with a request to join the study voluntarily. The e-mail contained a link to the research questionnaire and all necessary information about the study’s purpose and rules of participation. Moreover, potential respondents were able to download the link to the study questionnaire from the Poznan University of Medical Sciences website promoting our study project and social media (Facebook, LinkedIn). A total of 1,419 respondents (all who agreed to participate in the study) were taken into consideration. However, based on the inclusion criteria of the study and incomplete questionnaires by 289 healthcare workers, 1,130 respondents were finally included in the study. The response rate, defined as the number of adequately completed online forms, was 22%. Among respondents, 317 persons (28.05%) were unvaccinated (control group). To the study group, 813 vaccinated respondents (71.95%), including 76.88% women and 23.12% men, were recruited. Respondents were qualified based on the inclusion criteria, such as: age of consent, place of residence in Poland, giving voluntary and informed consent to participate in the study, access to the Internet, and being vaccinated at least with one dose of currently available SARS-CoV-2 vaccines. The exclusion criteria were as follows: age under 18 y., place of residence outside Poland, lack of consent to participate in the study, no Internet access, and not being vaccinated against COVID-19. Participants with reduced immunity and individuals with prior clinical or microbiological diagnosis of COVID-19 were excluded from the study. Given the observational and exploratory nature of the study, the sample size was pragmatic rather than hypothesis-driven.

The study was approved by the Bioethics Committee at the Poznan University of Medical Sciences in Poznań, Poland. In the document (as of November 24, 2021) there is a confirmation that the research project had no features of a medical experiment. Before enrollment, each volunteer was instructed about the study goals and informed about the safety and anonymity of the study. Volunteers were allowed to withdraw their consent to participate in the survey at any time without providing a reason. The obtained information was fully confidential. The identity of all surveyed people was anonymous, and no personal data of respondents were registered. Therefore, the research project did not violate the Personal Data Protection Act, as it did not use any data allowing for the subsequent identification of participants.

### 2.2 Research methods and time horizon

On the base of a literature review, focused group discussions, and expert opinions, a validated anonymous questionnaire was constructed with the use of Google Forms (25, 26). Time horizon of the study was from November 2021 to February 2022. Because of the government restrictions resulted from the state of pandemic, as well as in order to minimize the risk of SARS-Cov2 infection among respondents, the survey was conducted using the Computer-Assisted Web Interview method (25, 26). All study questionnaires were distributed online via institutional email invitations and through the university website and social media platforms. A non-probability convenience sampling strategy was used, with participants recruited voluntarily. To prevent multiple submissions from the same individual, the Google Forms platform was configured to recognize IP addresses. The study questionnaire contained mostly mandatory fields, which made it impossible to omit some answers. The questionnaire was developed following a standardized protocol that consisted of a literature review, focused group discussions, and an expert opinion. A pre-test procedure on a representative sample of 300 subjects (representative sample: 75 doctors, 75 nurses, 75 pharmacists and 75 students) was used as a suitable method for validation and for assessing the psychometric properties of the questionnaire. After the procedure, if necessary, the questions could be modified. However, the pre-test results were included in the post-test because the pre-test showed no need to modify the study tool. The internal consistency of the safety profile of COVID-19 vaccines questionnaire is based on the extent to which the items are correlated and was determined by calculating Cronbach’s *α*. Cronbach’s α was 0.73, which proves an excellent internal consistency. The study questionnaire was accepted by a group of 5 national medical consultants specializing in the field of infectious diseases.

The questionnaire comprised 25 questions divided into four categories:

- Demographic data (age, sex, height, weight, profession, and geographic region).
- Medical anamneses (chronic illnesses, health status, smoking, and alcohol consumption).
- COVID-19-related anamneses (type of vaccine, number of vaccine doses, dates of vaccine doses, previous infection, and diagnosis date).
- Vaccine efficacy (confirmed infection with the SARS-CoV-2 virus, the nature/course of COVID-19 illness, health complications after COVID-19 illness).

The primary outcome was the percentage of confirmed Sars-CoV-2 infections after vaccination.

The questionnaire is included as Supplementary file S1.

### 2.3 Statistical analysis

Counts and percentages were used to present categorical variables. The numerical data were shown as median and interquartile range (Me, Q_1_-Q_3_) since data did not follow the normal distribution (Shapiro-Wilks test). The comparison between vaccinated and unvaccinated cohorts were performed with the use of non-parametric Mann–Whitney test. The comparison concerning 4 typed of vaccines (BNT162b2 vs. mRNA-1273 vs. ChAdOx1 nCoV-19 vs. Ad26. COV2-S) was performed by Kruskal-Wallis test with the Dunn’s post-hoc tests. The Bonferroni correction for multiple comparisons was used. The comparison between two categorical features was assessed by chi-square test of independence. In case larger than 2×2 contingency tables showed statistical significance, we proceeded to construct all possible 2×2 tables to indicate between which vaccines we found significant differences. We labeled homogeneous groups with corresponding letters. For all tests a *p* value of less than 0.05 was considered significant. The statistical analysis was performed using the Statistica data analysis software v. 13.3 (TIBCO Software Inc. 2017, Palo Alto, United States).^1^

## 3 Results

### 3.1 The characteristics of all the surveyed respondents

There were 1,130 surveyed medical students and healthcare professionals. The comparative characteristics of all the respondents is presented in Table 1.

**Table 1:** Comparative characteristics of the surveyed respondents (*n* = 1,130).

Variables	Respondents		*p* value
	Unvaccinated respondents (control group)	Vaccinated respondents (study group)	
Group size *n* [%]	317 (28.05%)	813 (71.95%)	
Gender *n* [%]			
Women	235 [74.13]	625 [76.88]	0.3313^*^
Men	82 [25.87]	188 [23.12]	
Age [Median (Q1-Q3)]	32.00 (24.00–40.00)	30.00 (23.00–42.00)	0.2389^#^
Place of residence *n* [%]			
Village	98 [30.91]	207 [25.46]	<0.0001^*^
City up to 25,000 inhabitants	56 [17.67]	96 [11.81]	
City from 25 to 100 thousand inhabitants	47 [14.83]	110 [13.53]	
City with more than 100,000 inhabitants	57 [17.98]	137 [16.85]	
City with more than 500,000 inhabitants	59 [18.61]	263 [32.35]	
Education *n* [%]			
Secondary	121 [38.17]	311 [38.25]	0.1669^*^
Higher	196 [61.83]	502 [61.75]	
Self-estimated health status *n* [%]			
Excellent^&^	42 [13.22]	113 [13.85]	0.4156^*^
Very good	111 [35.13]	295 [36.26]	
Good	106 [33.22]	277 [34.12]	
Not so good	24 [7.62]	70 [8.58]	
Poor^	34 [10.81]	58 [7.19]	
Presence of comorbidities *n* [%]	127 [40.06]	334 [41.08]	0.5147^*^
Use of stimulants *n* [%]			
Alcohol	123 [38.80]	334 [41.08]	0.12834^*^
Nicotine	51 [16.08]	114 [14.02]	0.82153^*^
Alcohol and Nicotine	31 [9.77]	98 [12.05]	0.06931^*^

*The chi-square for independence; ^#^The U Mann–Whitney; ^&^overall physical, mental, and social well-being; ^^-^ complete absence of overall physical, mental, and social well-being.

Unvaccinated subjects (control group) statistically more often lived in villages and smaller cities (less than 100.000 inhabitants, *p* < 0.0001). To the final study group, 813 vaccinated subjects, including 625 women and 188 men, were recruited. Analyzed study groups: the control group and the experimental group did not significantly differ in terms of key socio-clinical characteristics: gender, age of participants, level of education, health status, presence of comorbidities, and substance use.

### 3.2 The characteristics of the study group

The sociodemographic characteristics of the vaccinated respondents (study group) is presented in Table 2.

**Table 2:** Comparative characteristics of the surveyed respondents vaccinated against the SARS-CoV-2 virus (*n* = 813).

Variable	Type of SARS-CoV-2 virus vaccine				*p* value
	BNT162b2 Pfizer/BioNTech (Group 1)	ChAdOx1 nCoV-19 (Oxford/AZ; Group 2)	mRNA-1273 Moderna (Group 3)	Ad26. COV2-S Johnson & Johnson (Group 4)	
Group size *n* [%]	543 (66.79%)	97 (11.93%)	91 (11.19%)	82 (10.09%)	
n [%]					
Women	433 [79.74]	71 [73.20]	63 [69.23]	58 [70.73]	0.04639^*^
Men	110 [20.26]	26 [26.80]	28 [33.77]	24 [29.27]	
Age [Median (Q1–Q3)]	27.00 (23.00–44.00)	32.00 (24.00–50.00)	28.00 (24.00–39.00)	29.00 (24.00–41.00)	<0.0001^#^
Self-estimated health status *n* [%]					
Excellent	64 [11.78]	10 [10.30]	9 [9.89]	8 [9.75]	0.16683^*^
Very good	217 [39.96]	39 [40.21]	36 [39.56]	35 [42.68]	
Good	202 [37.02]	36 [37.11]	33 [36.26]	31 [37.80]	
Not so good	30 [5.62]	10 [10.39]	10 [10.29]	6 [6.77]	
Poor	30 [5.62]	2 [2.00]	3 [4.00]	2 [3.00]	
Presence of comorbidities n [%]	261 [48.06]	47 [48.45]	38 [41.75]	37 [45.12]	0.3753^*^
Most common comorbidity types *n* [%]					
Heart failure	11 [2.02]	2 [2.76]	2 [2.19]	2 [2.43]	0.83208^*^
Obesity	32 [5.89]	5 [4.83]	9 [9.89]	5 [6.09]	0.22380^*^
Hypothyroidism	43 [7.92]^a^	3 [3.09] ^b^	5 [5.49]^a^	7 [8.53]^a^	0.04243^*^
Depression	21 [3.86]	3 [3.09]	4 [4.00]	3 [3.65]	0.47660^*^
Allergy	87 [16.02]	13 [13.40]	12 [13.33]	12 [14.63]	0.57027^*^
Arterial Hypertension	55 [10.13]^a^	11 [11.34]^a^	4 [4.67]^b^	6 [7.31]^a^	0.03212^*^
Diabetes	24 [4.42]	5 [4.83]	3 [3.33]	4 [4.87]	0.68041^*^
Time that has elapsed since receiving the last dose of the vaccine [months]	4.00 [1.00–5.00]	5.00 [4.00–6.00]	5.00 [1.00–5.00]	4.00 [2.00–5.00]	0.07748^#^
Use of stimulants *n* [%]					
Alcohol	218 [40.14]	32 [32.98]	40 [44.04]	33 [40.24]	0.14784^*^
Nicotine	71 [13.07]	12 [12.37]	13 [14.28]	10 [12.19]	0.82153^*^

*The chi-square for independence; ^#^The Kruskal-Wallis test; ^a,b^groups followed by the same letters do not differ statistically significantly.

Participants vaccinated with the BNT162b2 vaccine were the most numerous group (*n* = 543, 66.79%), 97 respondents (11.93%) were vaccinated with ChAdOx1 nCoV-19 vaccine, 91 people (11.19%) with the mRNA-1273 vaccine, while the Ad26. COV2. S vaccine was administered to the smallest group (82 people, 10.09%). There was no respondents vaccinated with newly introduced (January 13, 2022) the Nuvaxovid protein vaccine by NOVAVAX as it was virtually absent from the pharmaceutical market at the time of the study. The study group differ in terms of age, gender and selected comorbidities. Respondents vaccinated with mRNA-1273 were the youngest group, while those vaccinated with ChAdOx1nCoV-19 were the oldest one. Over two thirds of participants were females in each of 4 groups. There were statistically significant differences regarding the percentage of individuals with arterial hypertension and hypothyroidism.

### 3.3 The effectiveness of COVID-19 vaccines available in Poland

Table 3 shows the effectiveness of COVID-19 vaccines. Unvaccinated respondents significantly more often were infected with SARS-CoV-2. Mild and moderate courses of COVID-19 were more often declared by vaccinated subjects, while severe courses were more often observed among unvaccinated respondents.

**Table 3:** Evaluation of the effectiveness of COVID-19 vaccines available in Poland (*n* = 1,130).

Variables	Respondents		*p* value
	Unvaccinated respondent (control group; *n* = 317)	Vaccinated respondent (study group; *n* = 813)	
Confirmed infection with the SARS-CoV-2 virus n [%]	219 (69.08%)	70 (8.61%)	<0.00001^*^
The nature/course of COVID-19 illness *n* [%]			
Mild	70 [32.11]	32 [45.71]	0.00035^*^
Moderate	81 [37.16]	33 [47.14]	
Severe	67 [30.73]	5 [7.14]	

*The chi-square for independence.

As a result of the survey, it was found that the SARS-CoV-2 virus vaccines available on the market ensure high effectiveness in preventing its infection. The vast majority of 743 respondents (91.39%) had no confirmed COVID-19 infection after taking one of the vaccines available on the market. The rest of study population (70 individuals, 8.61%) were infected with SARS-CoV-2.

### 3.4 Incidence of COVID-19 depending on the type of vaccine taken

There was no statistically significant differences in the prevention of SARS-CoV-2 infection depending on the type of vaccine taken (*p* = 0.64). The largest percentage of respondents became infected after receiving the Johnson & Johnson vaccine (12.20%). The percentage of subjects with confirmed COVID-19 after being vaccinated with other preparations were as follows: the Pfizer/BioNTech vaccine 8.10%, the Oxford/AstraZeneca vaccine 9.28%, and the Moderna vaccine 7.69% (Table 4).

**Table 4:** Incidence of COVID-19 depending on the type of vaccine taken (*n* = 813).

Type of COVID-19 vaccine used	Participants infected with SARS-CoV-2 after vaccination	Participants non-infected with SARS-CoV-2 after vaccination
	Vaccinated subjects *n* (%)	Vaccinated subjects *n* (%)
Pfizer/BioNTech	44 (8.10)	499 (91.90)
Johnson & Johnson	10 (12.2)	72 (87.80)
Oxford/AstraZeneca	9 (9.28)	88 (90.72)
Moderna	7 (7.69)	84 (92.31)

Comirnaty mRNA vaccine (BNTI162b2) Pfizer/BioNTech, ‘COVID-19 Vaccine Janssen’ vector vaccine (Ad26. COV.2-S)—later name Janssen Jcovden (Johnson & Johnson), Vaxzevria vector vaccine (ChAdOx1 nCoV-19) from Oxford/AstraZeneca, Spikevax mRNA vaccine (mRNA1273) from Moderna.

The largest percentage of respondents were infected with the SARS-CoV-2 virus after taking one dose of the analyzed vaccines (10.34%). After taking two doses of the vaccine, 9.62% of the subjects became infected. The smallest percentage of infections was observed after taking three doses of the vaccine (5.73%). However, there was no significant relationship between the number of doses of the SARS-CoV-2 vaccine and the occurrence of coronavirus infections after their administration (*p* = 0.75).

### 3.5 The course of COVID-19 disease after vaccination and the type of COVID-19 vaccine used

Among 70 vaccinated participants infected with SARS-CoV-2, there were 32 (45.72%) with mild course of COVID-19, 33 (47.14%) with moderate course and 5 (7.14%) with severe course of the disease.

There was no relationship between the type of vaccine and the course of COVID-19 disease after vaccination (*p* = 0.62; Table 5).

**Table 5:** The course of COVID-19 disease after vaccination and the type of COVID-19 vaccine used.

	The course of COVID-19 disease after vaccination		
	Mild *n* [%]	Moderate *n* [%]	Severe *n* [%]
Type of COVID-19 vaccine used			
Pfizer/BioNTech	23 [52.27]	19 [43.18]	2 [4.55]
Johnson & Johnson	2 [20.00]	7 [70.00]	1 [10.00]
Oxford/AstraZeneca	4 [44.44]	4 [44.44]	1 [11.11]
Moderna	3 [42.86]	3 [42.86]	1 [14.29]

Comirnaty mRNA vaccine (BNTI162b2) Pfizer/BioNTech, ‘COVID-19 Vaccine Janssen’ vector vaccine (Ad26. COV.2-S)—later name Janssen Jcovden (Johnson & Johnson), Vaxzevria vector vaccine (ChAdOx1 nCoV-19) from Oxford/AstraZeneca, Spikevax mRNA vaccine (mRNA1273) from Moderna.

### 3.6 The relationship between the course of COVID-19 after vaccination and the number of vaccine doses

Among respondents vaccinated with one dose, the course of COVID-19 disease was usually of a moderate character (77.78%), whereas among participants who received two doses of the vaccine, the course was mild (43.75%) or moderate (50.0%). Among people vaccinated with three doses, COVID-19 was usually mild (75.92%). However, there was no significant relationship between the number of doses taken and the course of the COVID-19 disease (*p* = 0.12; Table 6).

**Table 6:** The relationship between the course of COVID-19 after vaccination and the number of vaccine doses (chi-square test for independence).

	The course of COVID-19 disease after vaccination		
	Mild *n* [%]	Moderate *n* [%]	Severe *n* [%]
The number of vaccine doses			
1	1 [11.11]	7 [77.78]	1 [11.11]
2	21 [43.75]	24 [50.00]	3 [6.25]
3	10 [76.92]	2 [15.38]	1 [7.69]

### 3.7 Health complications after COVID-19 and the type of vaccine taken

Among 70 respondents, who became infected with SARS-CoV-2 after being vaccinated against COVID-19, 29 people (41.43%) admitted to have health complications after COVID-19. The occurrence of health complications after COVID-19 were as follows: 71.43% in the group of previously vaccinated persons with Moderna preparation, 40.91% in the case of using Pfizer/BioNTech vaccines, 40.00% in the group vaccinated with Johnson & Johnson and 22.22% among respondents vaccinated with Oxford/AstraZeneca product. There was no statistically significant relationship between the type of vaccine taken and the frequency of health complications after COVID-19 disease (*p* = 0.26). Only 2.86% of the vaccinated subjects required hospitalization due to COVID-19 vs. 30.76% among unvaccinated subjects (*p* < 0.0001). It has been shown that the type of vaccine administered significantly statistically (*p* = 0.006) influenced the need for hospital treatment for COVID-19. In the presented study, only individuals vaccinated with the Johnson & Johnson vaccine required hospitalization, and there were no cases of hospitalization following infection with the coronavirus after receiving the Pfizer/BioNTech, Moderna, or Oxford/AstraZeneca vaccines.

## 4 Discussion

### 4.1 Effectiveness of COVID-19 vaccines

The outbreak of the pandemic, in addition to health consequences, both mental and physical, has inevitably affected other areas of life. It caused a significant regression of the economy, noticeable consequences on the labor market, education and society (27).

The development of COVID-19 vaccines has enabled society to gain immunity, thus limiting the spread of the disease and mitigating its course, reducing mortality. The economic value of the COVID-19 vaccine consists of the value of minimizing new cases of sick people, as well as the value of quickly resuming the production activities that underpin the economy. In the presented work, the effectiveness of vaccines against COVID-19 (Pfizer/BioNTech, Moderna, Oxford/AstraZeneca and Johnson & Johnson- available in Poland) was estimated by the impact of vaccines on selected medical aspects. During the study period, the delta variant of the SARS-CoV-2 (B.1.617.2) predominated (97.5%) in Poland. As of January 4, 2022, there were only 74 cases of confirmed infections with Omicron, which has begun to spread widely (28). All available vaccines were highly effective against the current SARS-CoV-2 variants (11–19).

The medical issue was to determine the impact of the vaccine type and number of doses on the course of COVID-19 infection. The data collected during the survey showed that only 8.61% of the respondents were infected with SARS-CoV-2 after receiving the COVID-19 vaccine. Subjects vaccinated with the Johnson & Johnson preparation most often became infected after receiving the vaccine (12.20%). However, compared to other preparations, the percentage of infected was similar: Oxford/AstraZeneca vaccine (9.28%); Pfizer/BioNTech vaccine (8.10%), Moderna vaccine (7.69%). There were no significant differences in the effectiveness of preventing coronavirus infection. It must be admitted that the obtained results did not allow to identify one vaccination regimen as better.

Another parameter evaluating the effectiveness of vaccines was the course of infection with SARS-CoV-2. Nearly 46% of the respondents described it as mild, about 47% as moderate, and only 7% of the respondents as severe. There was no significant statistical relationship between the type of vaccine taken and the course of COVID-19 disease.

The presented data clearly indicate the comparable effectiveness of vaccines available on the market in preventing COVID-19 infections. Fan et al. found that the available vaccines were sufficiently effective in preventing infection with the SARS-CoV-2 virus, however, in their opinion, vaccines based on mRNA technology (Moderna, Pfizer/BioNTech) were more effective than vector technology (Oxford/AstraZeneca, Johnson & Johnson) (28). Our results prove that the incidence of SARS-CoV-2 virus infection decreased with the adoption of the next dose of the vaccine. Only 5.73% of the subjects who took a booster dose were infected with coronavirus. In addition, the mildest course of the COVID-19 disease was found in this study group. The data obtained are consistent with the scientific literature. The significant impact of the number of doses taken on the risk of infection was described by Fan et al., who showed that the risk of COVID-19 infection after taking two doses of vaccination is 2.5 times lower than after taking only one dose (29).

In the presented study, the relationship between the type of vaccine and COVID-19 complications was also analyzed as a parameter of the effectiveness of vaccines against COVID-19. Most often, health complications were observed among those vaccinated with Moderna (71.43%). Among people vaccinated with other preparations, health complications were less common (Pfizer/BioNTech 40.91%; Johnson & Johnson 40.0%; Oxford/AstraZeneca 22.22%). Nevertheless, the observed differences were not statistically significant.

However, a significant relationship was found between the need for hospitalization as a result of COVID-19 disease and the type of previously received vaccine. Persons who received the Johnson & Johnson vaccine were hospitalized more often than respondents vaccinated with other preparations (Pfizer/BioNTech, Moderna, Oxford/AstraZeneca). Mohammed et al. described the effectiveness of selected types of vaccines and compared them to each other (30). The authors concluded that the Johnson & Johnson vaccine protects the most weakly against a severe course of the disease that required hospitalization. This is in line with our findings. However, Mohammed et al. also noticed that taking the Johnson & Johnson vaccine reduced hospitalization by 100% compared to unvaccinated people (30).

In the Spanish population, Pardo-Seco et al. demonstrated that the BNT162b2 vaccine (Pfizer/BioNTech) resulted in a significant reduction in COVID-19 infections in all age groups. Moreover, this type of vaccine has been shown to be responsible for reducing the need for hospitalizations due to severe COVID-19, hospitalizations in intensive care units and finally mortality (31). In another Spanish study, Martínez-Baz et al. showed that full vaccination reduces SARS-CoV-2 infections by 66%, symptomatic COVID-19 by 42% and hospitalization for COVID-19 by 72% (32). In this cited study, 58.0% participants were vaccinated with Pfizer/BioNTech, 37.9% with Oxford/AstraZeneca, and 4.1% with Moderna (32). Similar findings were also published by Pritchard et al. who reported that vaccination with the BNT162b2 (Pfizer/BioNTech) or Oxford/AstraZeneca preparations significantly reduced COVID-19 infections (33). In the Chinese population, Yang et al. observed that inactivated COVID-19 vaccines provided significant protection against SARS-CoV-2 Omicron variant infections (34).

All age groups appeared to be vulnerable to COVID-19 disease. In the presented study respondents were relatively at young age, what might be responsible for greater effectiveness of analyzed vaccines. It has been showed that age is one of the key factors defining the risk of COVID-19 and the effectiveness of vaccines. The older adults, due to the physiologically occurring reduced immunity (‘immunosenescence’) and accompanying frailty syndrome, are at greater risk of severe COVID-19 course (35, 36). It might be assumed that being at young age means having less severe comorbidities. Based on the literature, they are the individuals with the highest mortality due to COVID-19. The high-risk group, regardless of older age, includes people suffering from respiratory diseases, including: COPD (Chronic Obstructive Pulmonary Disease), pulmonary fibrosis, pulmonary embolism, cystic fibrosis; numerous cardiovascular diseases, including coronary artery disease, chronic heart failure and arterial hypertension. Other diseases, such as chronic kidney and liver diseases, diabetes, immune defects, morbid obesity, cancers, also are responsible for the increase of COVID-19 complications (37, 38). Rojas-Botero et al. proved that the effectiveness of COVID-19 vaccines in preventing hospitalization and death due to SARS-CoV-2 infection decreased with age, what was also confirmed by other researchers and explained by all above-mentioned factors: frailty, multimorbidity and immunosenescence (39–41).

It should be underlined that our findings are consistent with recent real-world evidence on COVID-19 vaccine effectiveness. In a large multicenter European cohort study among healthcare workers (ORCHESTRA project), Porru et al. demonstrated that booster doses significantly reduce the risk of both symptomatic and asymptomatic SARS-CoV-2 breakthrough infections (42). Similarly, Bianchi et al., in a retrospective cohort study, confirmed the effectiveness of the BNT162b2 mRNA vaccine in preventing SARS-CoV-2 infection and symptomatic disease during follow-up (43). Furthermore, long-term population-based data from France showed that mRNA COVID-19 vaccination is associated with favorable long-term outcomes, including no increase in all-cause mortality among vaccinated individuals (44).

Taken together, these real-world findings support our results, which indicate that COVID-19 vaccination is associated with a reduced risk of infection, milder disease course, and a substantially lower risk of hospitalization.

### 4.2 Study limitations and strengths

Assessing the impact of vaccination against COVID-19 (preparations: Pfizer/BioNTech, Moderna, Oxford/AstraZeneca, Johnson & Johnson) on selected medical aspects, the above data show the rationality of introducing the SARS-CoV-2 vaccination program in Poland. Further research is needed to evaluate the program from a long-term perspective.

The presented study is innovative, as it provides the first scientific data on the medical effectiveness of the COVID-19 vaccination program in Poland. To the best of our knowledge, only data on the attitude of surveyed Poles to SARS-CoV-2 vaccines and the safety profile of available preparations have been published (25, 45).

Even though this is a pioneering study in Poland testing the effectiveness of COVID-19 vaccines, there are some limitations. The primary weakness of this research relates to self-reported data rather than objective information from medical records. As the study was based on self-reported data, the possibility of recall bias and social desirability bias cannot be excluded. However, the strength of the analyzed data is that it was reported by healthcare professionals and medical students. Their knowledge and clinical practice allowed for much more objective data to be obtained than that of the general population.

The authors are aware that online research does not allow for random selection of respondents due to limited access to the Internet or the participant’s interest in the researched problem. The strength of an online survey is that it consist mainly of mandatory fields, which makes it impossible to miss some points, which is often the case with paper questionnaires. Gorrasi et al. even showed that when respondents complete an online survey they feel more anonymous and provide more reliable information, especially regarding health problems (46).

Another limiting factor that should be taken into account when analyzing the study results is vaccine hesitancy among healthcare workers. As mentioned above, among the 1,130 respondents, 317 (28.05%) were unvaccinated (control group), while 813 individuals (71.95%) were vaccinated. Potential reasons for not receiving vaccination may include general reluctance toward vaccines, lack of confidence in their effectiveness in controlling the pandemic, concerns about side effects, and medical contraindications. Similar determinants of vaccine hesitancy among healthcare workers have been reported in previous studies, including concerns about vaccine safety, mistrust, and insufficient knowledge (47, 48).

The proportion of unvaccinated healthcare workers in our study was higher than that reported in the Italian study by Gallé et al. (49). This finding highlights the importance of addressing vaccine hesitancy even among healthcare professionals, who serve as trusted sources of health information for the general population.

In this context, effective communication strategies are essential to improve vaccine uptake. Evidence suggests that targeted educational interventions, transparent risk communication, and the use of digital and social media platforms can significantly enhance vaccination acceptance (50, 51). Strengthening trust in public health institutions and addressing misinformation should be considered key components of vaccination campaigns. Without adequate acceptance and promotion of vaccination, SARS-CoV-2 may continue to pose significant public health challenges (52, 53).

When analyzing the limitation of the research, attention should be paid to the predominance of younger respondents. It may be related to easy access to the Internet and their willingness to participate in online surveys. This project was carried out during the COVID-19 pandemic, and a web-based study was a better solution, ensuring maximum safety for all study participants. However, this may have resulted in the exclusion of healthcare workers with limited access to the Internet.

10% of surveyed participants had their antibody titers tested after receiving the full series of vaccinations against the SARS-Cov-2 virus. Therefore, due to the low percentage of data (non-representativeness in relation to the entire study group), it was not included as a parameter for assessing the effectiveness of vaccines against COVID-19, which is also a limitation of the study.

Another limitation of the present study is the lack of multivariable analysis assessing determinants of vaccine uptake. Although the collected dataset would allow for such an analysis, the primary aim of this study was to evaluate vaccine effectiveness in terms of infection risk, disease course, complications, and hospitalization. Future studies should consider applying multivariable regression models to identify independent predictors of vaccination uptake among healthcare professionals and medical students.

Findings from this research are innovative and can be used in a global analysis of the effectiveness of the most commonly administered vaccines against COVID-19. Therefore, the presented study meets the information needs of decision-makers and healthcare professionals. Our results may constitute the basis for the developing clear recommendations regarding vaccination against SARS-CoV-2.

## 5 Conclusion

The vaccines available on the European market against the SARS-CoV-2 virus are effective in preventing infection and reducing the risk of severe progression of COVID-19. The type of preparation taken did not affect the re-infection with the coronavirus, its course and the risk of complications. Individuals receiving the Johnson & Johnson vaccine during an active infection were more likely to need hospitalization than those receiving the other vaccinations. Overall, vaccinated people were significantly less likely to be hospitalized due to COVID-19. There was no significant relationship between the number of doses of the SARS-CoV-2 vaccine and the occurrence of COVID-19 after their administration.

### 5.1 Future directions

In the future, our findings may support vaccination campaigns, as public willingness to be vaccinated remains a key determinant of their success (54, 55). Our results are consistent with previous studies showing that healthcare professionals serve as important role models and trusted sources of vaccination information for the general population (49, 56). Continued research and vigilant monitoring of vaccine effectiveness are essential, particularly as new variants emerge and vaccination strategies evolve (57).

Experts long predicted that widespread global vaccination would be critical to controlling the SARS-CoV-2 pandemic, a projection that has largely been confirmed (58, 59). Current recommendations highlight that COVID-19 vaccines and booster doses continue to provide strong protection against severe disease, including among those at highest risk (60). Retrospective analyses also demonstrate the long-term safety and effectiveness of SARS-CoV-2 vaccines, supporting their continued inclusion in clinical guidelines (61, 62).

## Data Availability

The raw data supporting the conclusions of this article will be made available by the authors, without undue reservation.

## Footnotes

1
http://statistica.io
