# Supplementary material for: COVID-19 vaccination remains essential for healthcare professionals: clinical evidence from a Polish cross-sectional study
Source: Front Public Health. 2026 Apr 16;14:1750819. doi: 10.3389/fpubh.2026.1750819 (PMC13128642; doi:10.3389/fpubh.2026.1750819)
Supplement: Supplementary file 1 [file Supplementary_file_1.docx]

**File S1 “Study questionnaire”**

**“Pharmacoeconomic aspects of the SARS-CoV-2 vaccination program in Poland”**

We encourage everyone over the age of 18 to complete the following questionnaire:

“Pharmacoeconomic aspects of the SARS-CoV-2 vaccination program in Poland”

**Dear Sir or Madam,**

Department of Internal Diseases, Metabolic Disorders and Arterial Hypertension as well as Department of Pharmacoeconomics and Social Pharmacy of the Poznan University of Medical Sciences are conducting a survey of the public opinion regarding the SARS-CoV-2 vaccination program in Poland.

The survey aims to collect information necessary for a scientific analysis and to improve the strategy of vaccinations against COVID-19.

The survey is safe, free of charge, and anonymous. Personal data of respondents shall not be recorded. Data that could be used to identify the survey participants in the future shall not be used.

Upon agreeing to participate in the survey, you will be asked to answer 33 questions in an online questionnaire, which will be used to collect the required demographic information, assess a history of your vaccinations against SARS-CoV-2, knowledge and personal attitude towards vaccination, and health-related information with regard to COVID-19.

Every respondent may approach the authors to ask for information regarding the survey questions and to obtain a report once the survey has been completed.

**Consent to take part in the survey**

I have read and understood the information pertaining to the survey. By responding to this questionnaire, I voluntarily consent to participate in the survey and I am aware that I can withdraw my consent to participate in the subsequent parts of the survey at any time without providing a reason.

Pursuant to the Polish law (Personal Data Protection Act of 10 May 2018), this survey shall be anonymous.

1. **Gender: ***

- Female
- Male

1. **Age: ***

- 18-24
- 25-40
- 41-60
- more than 60.

1. **Education: ***

- Vocational
- Primary
- Secondary
- Higher

1. **Your place of residence: ***

- Village
- Town, up to 25 thousand residents
- Town, more than 25 and up to 100 thousand residents
- City, more than 100 and up to 500 thousand residents
- City, more than 500 thousand residents.

1. **Have you ever been vaccinated against SARS-CoV-2 (virus responsible for COVID-19) ? ***

- Yes
- No - please go to question 18. The following questions only apply to SARS-CoV-2 vaccines (not other vaccines).

1. **How many doses have you been vaccinated? ***

- 1
- 2
- 3

1. **How long has it been since your last dose of vaccine? ***

- Less than a month
- One month
- Two months
- Three months
- Four months
- Five months
- Six months

1. **What vaccine did you take? (multiple choice question) ***

- BioNTech, Pfizer
- Johnson & Johnson
- Moderna
- Oxford, AstraZeneca

1. **Have you experienced any side effects after taking the vaccine ? ***

- Yes
- No - please go to question 14

1. **Which of the following side effects have you experienced after taking the SARS-CoV-2 vaccine? (multiple choice question) ***

- Pain or swelling at the injection site
- Fatique
- Headache
- Muscle pain
- Chills
- Joint pain
- Fever
- Other answer (please specify):

1. **How was the strength of side effects after vaccination against the SARS-CoV-2 ?**

- Mild
- Moderate
- Severe

1. **When did side effects occur after vaccination against SARS-CoV-2? (multiple choice question)**

- On the first day after vaccination
- On the second day after vaccination
- On day 3 after vaccination
- On day 4 after vaccination
- Other answer (please specify)

1. **After which dose of the SARS-CoV-2 vaccine did you experience side effects? (multiple choice question)**

- After first dose
- After second dose
- After third dose

1. **Have you been infected with coronavirus after you received the SARS-CoV-2 vaccine?**

- Yes
- No – please go to question 19.

1. **Have you had any health complications as a result of COVID-19 after you were vaccinated?**

- Yes
- No

1. **Did you have to be hospitalized as a result of COVID-19 after you were vaccinated?**

- Yes
- No

1. **What was the course of the COVID-19?**

- Mild
- Moderate
- Severe
- Extremely severe

**Please go to question 19.**

1. **What is the reason for not being vaccinated against SARS-CoV-2 ?**

- Fear of the risk of side effects and complications
- Concern about the ineffectiveness of the vaccine
- I have a disease that prevents me from being vaccinated
- I take medication that prevents me from being vaccinated
- Other answer (please specify): …

1. **Have you had a test for antibodies against SARS-CoV-2 ?**

- Yes
- No – please go to question 22

1. **What type of SARS-CoV-2 antibody test have you had?**

- IgG antibody detection test
- IgM antibody detection test
- IgG + IgM antibody detection test

1. **What antiboty level was detected in your test (please specify): ….**
2. **Are you a parent of a child aged 12-18?**

- Yes
- No – please go to question 29

1. **Is your child vaccinated against SARS-CoV-2 ?**

- Yes
- No – please go to question 28

1. **Has your child experienced any side effects after receiving the vaccine?**

- Yes
- No – please go to question 28

1. **Which of the following side effects did your child experience after receiving the SARS-CoV-2 vaccine? (multiple choice question)**

- Pain or swelling at the injection site
- Fatique
- Headache
- Muscle pain
- Chills
- Joint pain
- Fever
- Other answer (please specify):

1. **How was the strength of your child’s side effects after vaccination against the SARS-CoV-2 ?**

- Mild
- Moderate
- Severe

1. **When did your child experience side effects after vaccination against SARS-CoV-2? (multiple choice question)**

- On the first day after vaccination
- On the second day after vaccination
- On day 3 after vaccination
- On day 4 after vaccination
- Other answer (please specify)

**Please go to question 29.**

1. **What is the reason for not vaccinating your child (aged 12-18) against the SARS-CoV-2 virus?**

- Concern about the risk of side effects
- Concern about the ineffectiveness of the vaccine
- I am against vaccinations
- Lack of long-term studies on the safety of this vaccine
- Other answer (please specify): …

1. **Are you currently employed (paid work)?**

- Yes
- No – please go to question 33

1. **In the past twelve months, how many days did you miss work due to the COVID-19 pandemic? Please specify an approximate number of working days missed during the lockdown? (open question)**
2. **In the past twelve months, how many days did you miss work due to the COVID-19 pandemic? Please specify an approximate number of working days missed on sick leave due to your COVID-19 infection (open question)**
3. **Over the past twelve months, how much has the COVID-19 pandemic affected your performance at work? Think back to days when your work was limited in scope or type, when you achieved less than you wanted, or when you could not work as attentively as usual.**

**A single-choice question, in the form of a linear scale, where 1 means that: “The COVID-19 pandemic has not affected my work”, and 5: “The COVID-19 pandemic has completely prevented me from working.”**

**Mark your answer:**

- 1
- 2
- 3
- 4
- 5

1. **Over the past twelve months, to what extent has the SARS-CoV-2 pandemic affected your ability to perform daily activities outside of paid work? By daily activities we mean e.g. going around the house, shopping, taking care of a child, exercising, studying, etc. Please recall those times when your activities were limited in scope or type, or when you reached less than you wanted.**

**A single-choice question, in the form of a linear scale, where 1 means that: “The SARS-CoV-2 pandemic did not affect my daily activities”, and 5: “The SARS-CoV-2 pandemic completely prevented me from performing my daily activities”.**

**Mark your answer:**

- 1
- 2
- 3
- 4
- 5

*** Mandatory fields**
